# Effect of Selective Serotonin Reuptake Inhibitors and Immunomodulator on Cytokines Levels: An Alternative Therapy for Patients with Major Depressive Disorder

**DOI:** 10.1155/2013/267871

**Published:** 2013-11-17

**Authors:** María Eugenia Hernandez, Danelia Mendieta, Mayra Pérez-Tapia, Rafael Bojalil, Iris Estrada-Garcia, Sergio Estrada-Parra, Lenin Pavón

**Affiliations:** ^1^Department of Psychoimmunology, National Institute of Psychiatry “Ramón de la Fuente”, Calzada México-Xochimilco 101, Col. San Lorenzo Huipulco, Tlalpan, 14370 Mexico City, DF, Mexico; ^2^Universidad Autónoma Metropolitana. Avenida San Rafael Atlixco No. 186, Col. Vicentina, Iztapalapa, 09340 Mexico City, DF, Mexico; ^3^National Institute of Psychiatry “Ramón de la Fuente”, Calzada México-Xochimilco 101, Col. San Lorenzo Huipulco, Tlalpan, 14370 Mexico City, DF, Mexico; ^4^Department of Immunology, National School of Biological Sciences (ENCB), National Polytechnic Institute (IPN), 11340 Mexico City, DF, Mexico; ^5^Departamento de Inmunología, Instituto Nacional de Cardiología, 14080 Mexico City, DF, Mexico

## Abstract

Major depressive disorder (MDD) is a psychiatric illness that presents as a deficit of serotonergic neurotransmission in the central nervous system. MDD patients also experience alterations in cortisol and cytokines levels. Treatment with selective serotonin reuptake inhibitors (SSRIs) is the first-line antidepressant regimen for MDD. The aim of this study was to determine the effect of a combination of SSRIs and an immunomodulator—human dialyzable leukocyte extract (hDLE)—on cortisol and cytokines levels. Patients received SSRIs or SSRIs plus hDLE. The proinflammatory cytokines IL-1**β**, IL-2, and IFN-**γ**; anti-inflammatory cytokines IL-13 and IL-10; and 24-h urine cortisol were measured at weeks (W) 0, 5, 20, 36, and 52 of treatment. The reduction in cortisol levels in the SSRI-treated group was 30% until W52, in contrast, the combined treatment induced a 54% decrease at W36. The decline in cortisol in patients who were treated with SSRI plus hDLE correlated with reduction of anti-inflammatory cytokines and increases levels of proinflammatory cytokines at the study conclusion. These results suggest that the immune-stimulating activity of hDLE, in combination with SSRIs, restored the pro- and anti-inflammatory cytokine balance and cortisol levels in depressed patients versus those who were given SSRIs alone.

## 1. Introduction

Clinical and epidemiological studies have established that major depressive disorder (MDD) is a cause of chronic stress [1, 2]. The World Health Organization asserts that MDD will be the second leading cause of incapacity worldwide by 2030 [3], representing a tremendous public health problem with high economic costs [4]. Increased stress levels affect the duration and extent of symptoms of depression [5]. One of the most common clinical features of MDD is the development of hypothalamic-pituitary-adrenal (HPA) axis abnormalities [6, 7]. Chronic hyperactivity of the HPA axis induces hypercortisolism, which affects the nervous, endocrine, and immune systems [8]. The HPA axis function is upregulated by proinflammatory cytokines through the brain receptors for this soluble molecules, expressed mostly at hypothalamus [9]. This stimulation induces a rise on circulatory levels of glucocorticoids that decreases the inflammatory systemic effects induced by cytokines and diminishes the release of CRH at hypothalamus, generating a negative feedback loop.

Various studies have linked variations in cytokine and cortisol levels in MDD [10]; one of the first studies of the neuromodulatory effects of cytokines reported the induction of depressive symptoms by therapeutic IL-2 and IFNs [11]. Proinflammatory cytokines, such as IL-1*β*, IL-6, and TNF-*α*, also elicit adverse behavioral effects (fatigue, soporific effects) and symptoms of anxiety and depression [12].

MDD patients have high levels of cortisol in bodily fluids, such as saliva, blood, cerebrospinal fluid, and urine [13–15]. In addition, patients with MDD also experience deficits in serotonergic neurotransmission in the central nervous system [16], which is why they are treated successfully with selective serotonin reuptake inhibitors (SSRIs). 

SSRIs are designed to compensate for alterations in serotonin levels (5-HT) and are usually administered over 1 year [17]. The HPA axis is restored, following clinical responses to treatment with SSRIs—SSRIs decrease circulating cortisol levels by reestablishing the downregulated glucocorticoid receptor, increase serotonin levels in circulation by blocking its reuptake receptor (5-HTT), and modify circulating cytokine profiles by receptors for neurotransmitters, glucocorticoids, and cytokines [15, 18, 19]. 

Our group has reported that without pharmacological intervention, MDD patients have a predominantly anti-inflammatory cytokine profile that is associated with high cortisol levels [20, 21] and that the administration of SSRIs for 52 weeks reverses the symptoms of MDD and modifies the cortisol and cytokines altered levels without restoring to normal levels [20]. 

Controlled trials have reported that 30% to 40% of MDD patients become resistant to pharmacological treatments due to medical comorbidities, unavailability of appropriate services, and poor adherence to therapies [22]. Despite advances in SSRIs, the management of MDD still requires pharmacological modalities that restore the immunological and endocrine imbalance in depressed patients. 

Although the mechanisms of action are not fully understood, we used a dual pharmacological therapy SSRI plus human dialyzable leukocyte extracts (hDLEs) and measured cortisol and cytokines levels for 52 weeks of followup in depressed patients. hDLEs comprise many peptide sequences below 17 kDa [23], and hDLEs have been used widely as an adjuvant for patients with infectious diseases and deficient cell-mediated immune responses [24, 25]. DLEs stimulate the production of proinflammatory cytokines, including TNF-*α* and IL-6 [26], but their effects on the endocrine and immune dysfunction in MDD patients are unknown. This study examined the efficacy of hDLEs in reverting endocrine and immune alterations in adult outpatients with MDD. 

## 2. Materials and Methods

### 2.1. Patients

The outpatient clinic of Instituto Nacional de Psiquiatria “Ramón de Fuente,” Mexico City, assessed 434 individuals and recruited 65 Mexican patients. Patient recruitment was made according to the clinical experimental procedures set out in the INPRF-NC092318.0 research protocol, approved by the ethics committee of the Instituto Nacional de Psiquiatría, México. All subjects were diagnosed by psychiatrists who applied the Mini-International Neuropsychiatric Interview, a standardized diagnostic interview that is based on DSM-IV-TR criteria. Clinical status was measured using the Hamilton Depression Scale (HDRS) and Beck Depression Inventory (BDI). Patients who met the inclusion criteria were free of antidepressants for at least 3 weeks before the study. Each subject underwent laboratory screens to rule out other medical illnesses. After receiving a detailed explanation of the study aims, all participants signed written consent forms.

**Figure 1:**
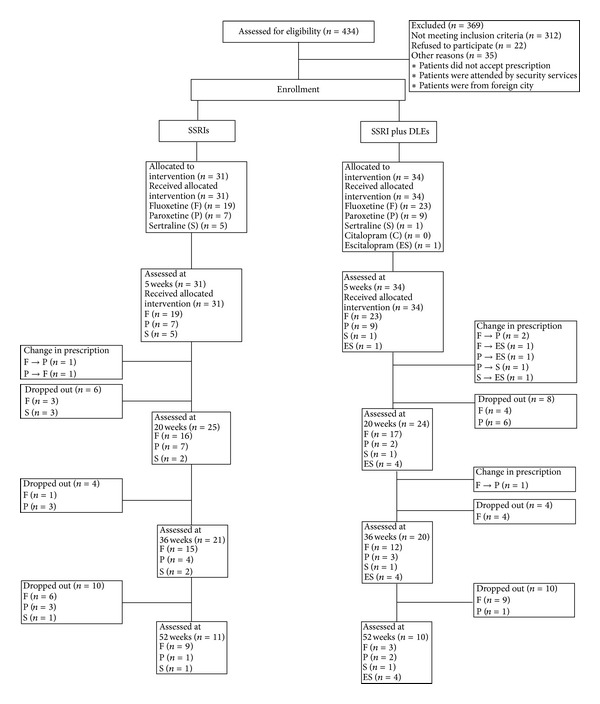
Flowchart of 52 week SSRIs and SSRIs plus hDLEs treatment in MDD patients. The numbers in parenthesis refer to the number of patients evaluated throughout the study, the changes in patient numbers for a pharmacological treatment, and the changes in patient numbers for treatment types withdrawn from the protocol. Change in prescription refers to the symbol >.

All patients were administered SSRIs (19 fluoxetine, 7 paroxetine, and 5 Sertraline) or SSRIs plus hDLEs (23 fluoxetine, 9 paroxetine, 1 sertraline, and 1 escitalopram). All patients were evaluated monthly by their psychiatrist, based on the HDRS and BDI. Blood and urine samples were obtained at weeks (W) 0, 5, 20, 36, and 52 of treatment.  Figure 1 shows the total number of patients who were evaluated throughout the study, the changes in pharmacological treatment, and the causes for protocol withdrawal. The patients' demographics are shown in Table 1.

### 2.2. Drugs

The doses of SSRIs (mg/day) were as follows: fluoxetine, 20; paroxetine, 20; sertraline, 100; and escitalopram, 10. SSRI doses were established for each patient by his physician and adjusted when it was necessary throughout the study; the drugs were paid for by the patients (Figure 1). 

hDLEs (Tranferon) were kindly provided by the Proyecto Factor de Transferencia, Escuela Nacional de Ciencias Biológicas, Instituto Politecnico Nacional (Mexico City, MX). For the group that was given SSRIs plus hDLEs, 30 units of hDLEs were administered to each patient for the 52 weeks of followup as follows: 3 units in week 1, 2 units in week 2, 1 unit each in weeks 3 and 4, and 1 unit every 2 weeks from Week 5 to the end of the study, as described [27].

### 2.3. Sample Collection and Measurement of Cortisol and Cytokines by RIA and ELISA

Participants were instructed to collect their urine for 24 hours, in which total cortisol was measured by radioimmunoassay (RIA). Circulatory levels of IL-1*β*, IL-2, IFN-*γ*, IL-4, IL-10, and IL-13 were measured in serum from 30 mL of peripheral blood. Blood and 24 h urine samples were collected at W0, W5, W20, W36, and W52.

### 2.4. ELISA of Cytokines

Human IL-1*β*, IL-2, IFN-*γ*, IL-4, IL-10, and IL-13 were measured by ELISA. The ranges of detection were (pg/mL): IFN-*γ*, 5–1000; IL-1*β*, 3.91–250; IL-2, 78–1250; IL-4, 31.25–2000; IL-10, 4–2000; and IL-13, 312–5000. Primary antibodies were diluted to 4 *μ*g/mL (except for anti-IL-10 and anti-IFN-*γ*; 8 *μ*g/mL), 100 *μ*L of which was added to each well of a 96-well plate. 

Nonspecific binding was prevented with 3% bovine serum albumin (BSA) in phosphate-buffered saline (PBS). Standards and samples were pipetted, and secondary antibodies were added to the plate. The secondary antibody concentrations were as follows (ng/mL): IFN-*γ*, 125; IL-1*β*, 100; IL-2, 25; IL-4, 50; IL-10, 400; and IL-13, 400. The immunoreactions were visualized with streptavidin-peroxidase solution using tetramethylbenzidine as substrate. The colorimetric reaction was stopped with sulfuric acid, and optical density was measured on a spectrophotometer (*λ* = 492 nm). The intra- and interassay variability was less than 5% and 7%, respectively.

### 2.5. Statistical Analysis

Data were analyzed using Prisma 6 for Mac OS X (GraphPad Software, La Jolla, CA, USA, http://www.graphpad.com/). Differences between means were analyzed using the homogeneity of variance test, followed by one-way ANOVA with Bonferroni's post hoc correction. Significant differences were calculated by comparing patients before antidepressant treatment (W0) and the healthy volunteers (HVs). Then, the values before antidepressant treatment (W0) were compared with those during the treatment (W5, W20, W36, and W52) in MDD patients. Finally, the data between patients at W52 and the HV were compared. Statistical significance was set to *P* < 0.05. 

## 3. Results

### 3.1. Clinical and Psychiatric Assessment

Clinical and laboratory parameters, as measured by the Institute's clinical laboratory, such as complete blood count, blood chemistry, thyroid function test (T3, T4, and TSH), and complete urinalysis, fell within normal ranges of reference values in MDD patients and healthy volunteers; no parameter differed significantly between groups (data not shown). Table 1 shows the demographics and data on recurrence for the study participants, and Table 2 shows the scores on the psychiatric scales. At W0, MDD patients had an HDRS score of (SSRIs = 20 ± 2 and SSRIs plus hDLEs = 24 ± 4 points). Clinical remission attained at W20, at which point the HDRS score was SSRIs (3.3 ± 2 points) and SSRIs plus hDLEs (2.8 ± 3 points), and was maintained until the end of the study.

### 3.2. Cortisol

**Figure 2:**
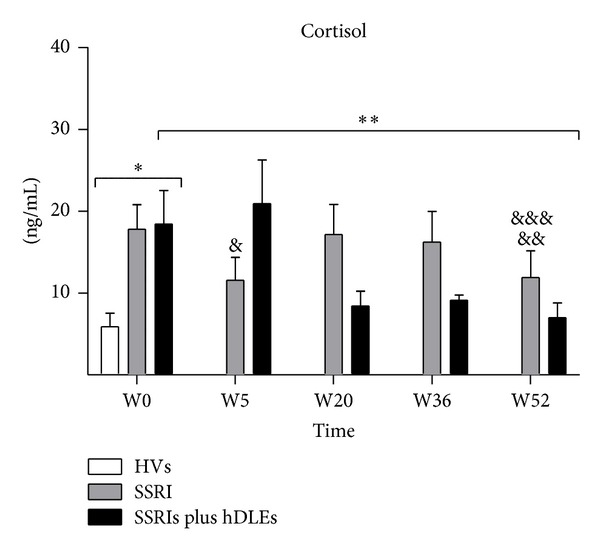
24 h urinary cortisol levels detected by RIA in healthy volunteers and MDD patients. Patients were treated with SSRIs or SSRIs plus hDLEs during 52 weeks of study. The statistical analyses were as follows. First, the patient group before antidepressant treatment (W0) was compared with the control group (HVs). Second, the values before the antidepressant treatment (W0) were compared with those during the treatment (W5, W20, W36, or W52) in patients. Third, the data of patients at W52 versus HVs were compared. *P < 0.0001, significant difference before treatment versus HVs. **P < 0.001 between SSRIs plus hDLEs treatment at W5, W20, W36, and W52 versus W0. ^&^ *P* < 0.0001 between SSRIs treatment at W5 versus W0. ^&&^ *P* < 0.05 between SSRIs treatment at W52 versus W0. ^&&&^ *P* < 0.05 between SSRIs treatment at W52 versus HVs. Data are expressed as mean ± SD. HVs: healthy volunteers; MDD: major depression disorder; W: weeks.

The concentrations of urinary cortisol in healthy volunteers and depressed patients before and throughout the 52 weeks of treatment are shown in Figure 2. Cortisol levels showed significant changes (*F*_[1,10]_ = 50.7, *P* < 0.0001). In MDD patients before treatment (W0) the hormone levels were significantly higher (SSRIs = 18 ± 3 and SSRIs plus hDLEs = 18 ± 4) than in healthy volunteers (6 ± 2). By post hoc comparison showed differences during treatments (SSRIs = 12 ± 3 and SSRIs plus hDLEs = 21 ± 5) at W5, (SSRIs = 17 ± 4 and SSRIs plus hDLEs = 8.5 ± 1.8) at W20, (SSRIs = 18 ± 4 and SSRIs plus hDLEs = 9 ± 0.6) at W36, and (SSRIs = 12 ± 3 and SSRIs plus hDLEs = 7 ± 1.8) at W52, when compared with W0 higher (SSRIs = 18 ± 3 and SSRIs plus hDLEs = 18 ± 4). At the end of the study, the cortisol level in MDD patients with SSRIs plus hDLEs treatment was not significantly different from that in the HVs. In contrast, the cortisol levels in MDD patients with SSRIs treatment showed a significant reduction when compared with healthy volunteers (Figure 2).

### 3.3. Proinflammatory Cytokines Profile

**Figure 3:**
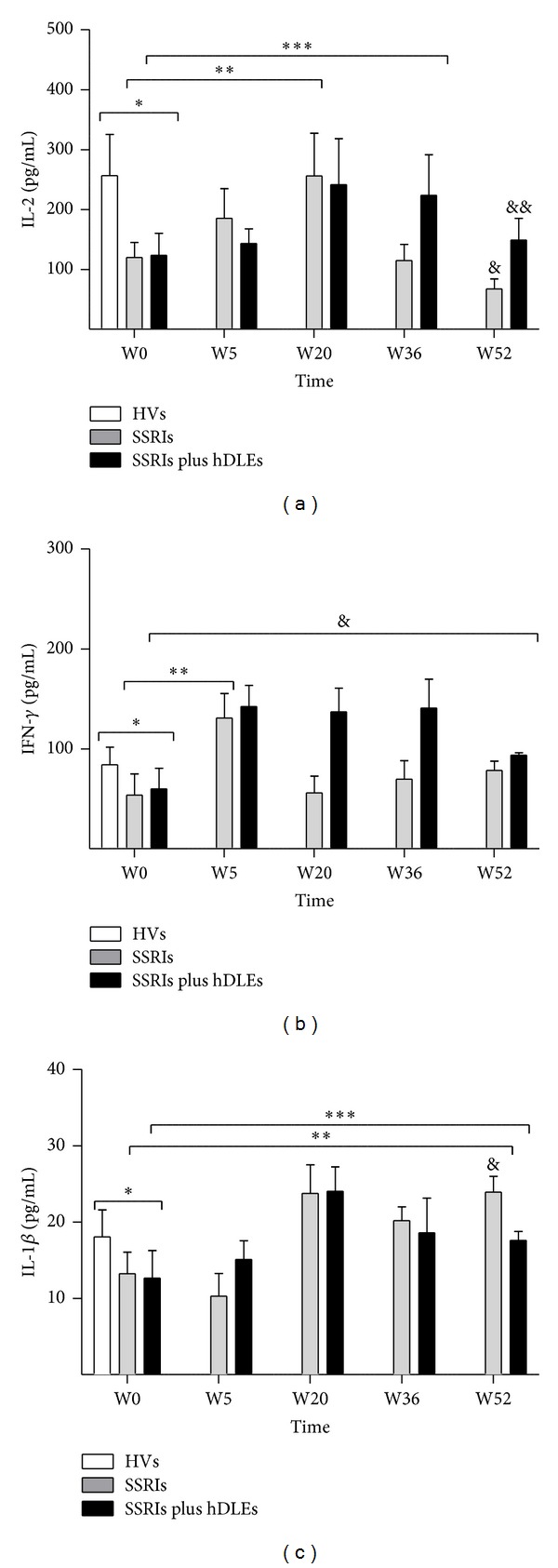
Serum proinflammatory cytokines detected by capture ELISSA assay in HVs and MDD patients. Patients were treated with SSRIs or SSRIs plus hDLEs during 52 weeks of study. The statistical analyses were as follows. First, the patient group before antidepressant treatment (W0) was compared with the control group (HVs). Second, the values before the antidepressant treatments (W0) were compared with those during the treatment (W5, W20, W36, or W52) in patients. Third, the data of patients at W52 versus HVs were compared. IL-2 (a), IFN-*γ* (b), and IL-1*β* (c): *P < 0.0001, significant difference before treatments versus HVs. IL2: **P < 0.0001 between SSRIs treatment at W5 and W20 versus W0. ***P < 0.001 between SSRIs plus hDLEs treatment at (W) 5, 20, and 36 versus W0. ^&^ *P* < 0.0001 between SSRIs treatment at W52 versus HVs. ^&&^ *P* < 0.001 between SSRIs plus hDLEs treatment at W52 versus HVs. IFN-*γ*: **P < 0.0001 between SSRIs treatment at W5 versus W0. ^&^ *P* < 0.0001 between SSRIs plus hDLEs treatment at (W) 5, 20, 36, and 52 versus W0. IL-1*β*: **P < 0.001 between SSRIs treatment at (W) 5, 20, 36, and 52 versus W0. ***P < 0.0001 between SSRIs plus hDLEs treatment at (W) 5, 20, 36, and 52 versus W0. ^&^ *P* < 0.0001 SSRIs treatment at W52 versus HVs. Data are expressed as mean ± SD. HVs: healthy volunteers; MDD: major depression disorder; W: weeks.

Variations in circulating proinflammatory cytokine levels were determined by ELISA using antibodies against cytokines specific. The levels expressed in pg/mL are shown in Figure 3(a) (IL-2), Figure 3(b) (IFN-*γ*), and Figure 3(c) (IL-1*β*).

#### 3.3.1. IL-2

IL-2 showed significant differences between the HVs and MDD patients before and during the SSRIs and SSRIs plus hDLEs treatments (*F*_[1,10]_ = 30.3, *P* < 0.0001). Prior to treatment, MDD patients had decreased IL-2 levels when compared with the HVs (SSRIs = 120 ± 25, SSRIs plus hDLEs = 123 ± 37 versus HV = 256 ± 69). MDD patients showed increases at W5 (SSRIs = 185 ± 49 and SSRIs plus hDLEs = 142 ± 24), W20 (SSRIs = 256 ± 71 and SSRIs plus hDLEs = 241 ± 71), W36 (SSRIs plus hDLEs = 224 ± 68), and W52 (SSRIs plus hDLEs = 149 ± 36). In contrast, IL-2 was significantly decrease with SSRIs (115 ± 27) at W36 and W52 (67 ± 16) (Figure 3(a)).

#### 3.3.2. IFN-*γ*

IFN-*γ* levels fluctuated during the treatments and showed significant changes (*F*_[1,10]_ = 66.9, *P* < 0.0001). Before treatment, patients had lower IFN-*γ* levels (SSRIs = 54 ± 21 and SSRIs plus hDLEs = 60 ± 21) compared with healthy volunteers (84 ± 17). At W5 (SSRIs = 131 ± 24 and SSRIs plus hDLEs = 142 ± 21), W20 (SSRIs = 66 ± 16 and SSRIs plus hDLEs = 137 ± 24), W36 (SSRIs = 69 ± 18 and SSRIs plus hDLEs = 141 ± 28), and W52 (SSRIs = 78 ± 9 and SSRIs plus hDLEs = 94 ± 3). At the end of treatment, IFN-*γ* levels were comparable with those of healthy volunteers (Figure 3(b)).

#### 3.3.3. IL-1*β*

IL-1*β* showed contrast and significant changes (F_[1,10]_ = 56.2, *P* < 0.0001). Before treatment, circulating levels of IL-1*β* were significantly lower in patients (SSRIs = 13 ± 3 and SSRIs plus hDLEs = 13 ± 4) versus healthy volunteers (18 ± 4). At W5, IL-1*β* increased with SSRIs plus hDLEs (15 ± 2) and fell with SSRIs (10 ± 3) compared to W0. Both treatments showed increases at W20 (SSRIs = 24 ± 4 and SSRIs plus hDLEs = 24 ± 3), W36 (SSRIs = 19 ± 2 and SSRIs plus hDLEs = 22 ± 1), and W52 (SSRIs = 19 ± 4 and SSRIs plus hDLEs = 18 ± 1). At the end of the followup, IL-1*β* levels of patients were comparable with those of healthy volunteers (Figure 3(c)).

### 3.4. Anti-Inflammatory Cytokines Profile

**Figure 4:**
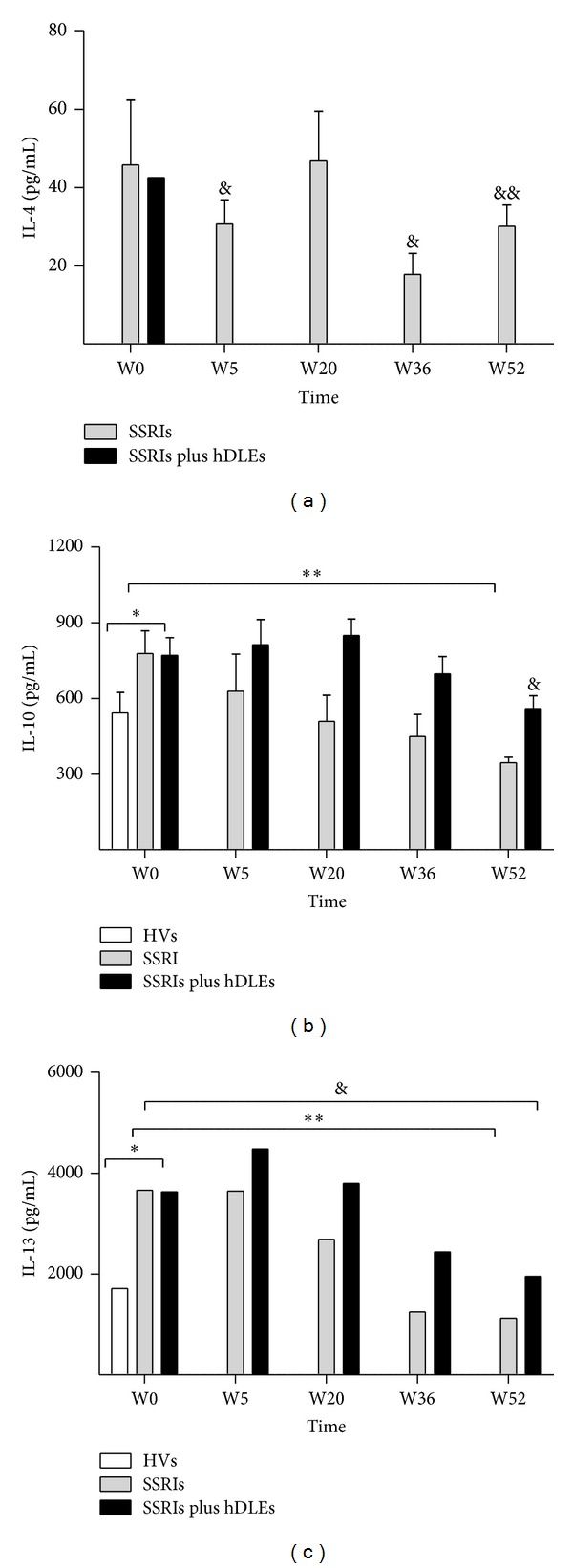
Serum anti-inflammatory cytokines detected by capture ELISSA assay in HVs and MDD patients. Patients were treated with SSRIs or SSRIs plus hDLEs during 52 weeks of study. The statistical analyses were as follows. First, the patient group before antidepressant treatment (W0) was compared with the control group (HVs). Second, the values before the antidepressant treatments (W0) were compared with those during the treatment (W5, W20, W36, or W52) in patients. Third, the data of patients at W52 versus HVs were compared. IL-4 (a), IL-10(b), and IL-13 (c). HVs were not detectable. IL-4: ^&^ *P* < 0.0001 SSRIs treatment at (W) 5 and 36 versus W0. ^&&^ *P* < 0.001 between SSRIs treatment at W52 versus W0. IL-10 and IL-13: *P < 0.0001, significant difference before treatments versus HVs. IL-10: **P < 0.001 between SSRIs treatment at (W) 5, 20, and 36 versus W0. ^&^ *P* < 0.0001 between SSRIs plus hDLEs treatment at W52 versus HVs. IL-13: **P < 0.001 between SSRIs treatment at (W) 5, 20, and 36 versus W0. ^&^ *P* < 0.0001 between SSRIs plus hDLEs treatment at (W) 5, 20, 36, and 52 versus W0. Data are expressed as mean ± SD. HVs: healthy volunteers; MDD: major depression disorder; W: weeks.

Variations in circulating anti-inflammatory cytokine levels are shown in Figure 4(a) (IL-4), 4B (IL-10), and 4C (IL-13). 

#### 3.4.1. IL-4

The values of circulating levels of IL-4 in healthy volunteers were below the range of sensitivity of the assay (31.25–1000 pg/mL). Thus, we considered these values nondetectable (ND). Before treatments, the patients showed significant increases in IL-4 (SSRIs = 45 ± 16 and SSRIs plus hDLEs = 42 ± 17). Changes in IL-4 levels fluctuated during treatments (*F*_[1,5]_ = 18, *P* < 0.0001). Patients treated with SSRIs had increased cytokine levels at followup study. In contrast, SSRIs plus hDLEs treatment showed values ND (Figure 4(a)).

#### 3.4.2. IL-10

IL-10 differed significantly before and during treatments (*F*_[1,10]_ = 63.6, *P* < 0.0001). This cytokine showed increase (SSRIs = 633 ± 84 and SSRIs plus hDLEs = 812 ± 100) at W5, W20 (SSRIs = 513 ± 151 and SSRIs plus hDLEs = 849 ± 65) versus W0 (SSRIs = 766 ± 84 and SSRIs plus hDLEs = 770 ± 69) and HVS (527 ± 99). In contrast, IL-10 levels declined at W36 (SSRIs = 450 ± 94 and SSRIs plus hDLEs = 697 ± 67) and W52 (SSRIs = 347 ± 31 and SSRIs plus hDLEs = 527 ± 99). At the end of the followup study, IL-10 levels of SSRIs group showed levels comparable with the healthy volunteers (Figure 4(b)).

#### 3.4.3. IL-13

Before treatment (W0), patients have significantly higher IL-13 levels than healthy volunteers (SSRIs = 3725 ± 708 and SSRIs plus hDLEs = 3630 ± 799 versus HVs = 1633 ± 172). IL-13 differed significantly during treatments (*F*_[1,10]_ = 115.3, *P* < 0.0001). Variations in cytokine levels showed at W5 (SSRIs = 3779 ± 380 and SSRIs plus hDLEs = 4437 ± 505), W20 (SSRIs = 2967 ± 443 and SSRIs plus hDLEs = 3793 ± 579), W36 (SSRIs = 1239 ± 245 and SSRIs plus hDLEs = 2438 ± 57), and W52 (SSRIs = 1116 ± 268 and SSRIs plus hDLEs = 1953 ± 271) versus W0 (SSRIs = 3725 ± 708 and SSRIs plus hDLEs = 3630 ± 799) (Figure 4(c)).

## 4. Discussion

### 4.1. Cortisol

Hyperactivity of the HPA axis in response to increased stress is linked to dysregulation of cortisol and serotonin secretion in various psychiatric disorders, such as major depression disorder [7, 28]. Cortisol levels are regulated by a negative feedback system of glucocorticoid receptors (GRs). GR is a steroid-activated nuclear receptor that, on binding to cortisol, translocates to the nucleus, where it targets genes that mediate cortisol and cytokine secretion and neuronal metabolism and plasticity [29]. 

Hypercortisolemia is common in MDD patients—clinical assays have reported higher cortisol levels in saliva, plasma, and cerebrospinal fluid in depressed patients who have not received pharmacological treatment [30, 31]. Before being administered SSRIs and hDLEs, our patients also experienced hypercortisolemia. Failure of the HPA axis to regulate circulating cortisol levels has been suggested to affect desensitization to GRs during stress and the inability to return to resting conditions, prolonging GR activation and its downstream effects [28]. 

These findings could explain the hypercortisolemia in patients with MDD, a common comorbidity in this disorder that is regarded as a marker of axis hyperactivity HPA [28]. Further, corticosteroids have a significant function in the link between stress and mood alterations, interacting with serotonin receptors (5-HRT_1A_, 5HT_2_) [32–34].

SSRIs, the most widely used antidepressants, upregulate extracellular serotonin concentrations by acutely blocking the serotonin transporter 5-HTT. 5-HTT regulates extracellular serotonin concentrations by removing 5-HT from the synaptic cleft [29]. Various clinical studies, including ours, have reported a decline in cortisol levels in fluids of MDD patients who have been treated with SSRIs [20, 35, 36]. After 1 year of treatment, however, cortisol levels are not comparable to healthy subjects [20]. 

This study examined the ability of coadministration of hDLEs and SSRI in MDD patients to restore cortisol and cytokine imbalances versus SSRIs alone. Our data show that, in depressed patients who were treated with SSRIs plus hDLE, cortisol levels, fell (54%) from W20 to the end of treatment. In contrast, SSRIs alone decreased such levels from W36 to W52. Notably, those who were given SSRIs plus hDLEs showed cortisol levels more nearby to healthy volunteers. 

The underlying molecular mechanism by which SSRIs function is unknown. 5-HTT controls the reuptake of serotonin from the synapse, and its inhibition by SSRIs increases serotonin levels at the synapse. However, SSRIs may influence the endocrine and immune systems of depressed patients. *In vitro *studies have demonstrated that SSRIs enhance GR-mediated transcription in the presence of cortisol [37] and have proposed that antidepressants inhibit membrane-bound steroid transporters, increasing the intracellular concentrations of the glucocorticoids [18], in turn enhancing GR expression and function and restoring the negative feedback by cortisol [38, 39]. 

Serotonin stimulates the secretory activity of the adrenal glands through 5-HT4 receptors [40, 41]. Moreover, glucocorticoid receptor antagonists increase the response to SSRIs with elevated serotonin levels [42, 43]; in contrast, exogenous administration of SSRIs decreases cortisol secretion *in vitro* [44]. The early and significant decline in cortisol levels in MDD patients who have been treated with SSRIs and hDLEs suggests that this combination enhances the effect of SSRIs on cortisol levels. 

### 4.2. Pro- and Anti-Inflammatory Cytokines

SSRIs also target cells of the immune system. A wide range of cytokine-producing cells constitutively express cortisol and the serotonin receptors 5-HT_1A,_ 5-HT_2A,_ 5-HT_1B,_ and 5-HT_3_ [29, 45, 46]. Serotonin receptors activate cAMP-dependent pathways and mediate synthesis and release of cytokines and cellular proliferation [9]. Cortisol modulates cytokine gene transcription and lymphocyte proliferation. Studies of dexamethasone have reported alterations in GR in leukocytes of depressed patients, such as decreased nuclear translocation [47] and cellular proliferation [48]. In addition, lymphocytes from MDD patients have a lower density of 5-HTT [49] and impaired 5-HT_1A_ receptor functions [45, 46]. 

Variations in cortisol and serotonin concentrations can directly affect cytokine-producing cells and modulate the pattern of cytokine release [50]. We have reported an anti-inflammatory cytokine profile in MDD patients before pharmacological treatment [20, 21]. In this study, MDD patients had significantly higher IL-4, IL-10, and IL-13 levels at W0 versus healthy volunteers and lower proinflammatory cytokine levels (IL-1*β*, IFN-*γ*, and IL-2). 

The immune system responds to stressful stimuli by secreting proinflammatory cytokines [51], but when the rise in stress becomes chronic, the cytokines that are produced by immune cells activate the HPA axis, stimulating the adrenal cortex to synthesize and release glucocorticoids, which ultimately suppress proinflammatory gene expression [34]. For example, glucocorticoids upregulate IL-4, IL-10, and IL-13 production and can induce immunosuppression with higher and sustained glucocorticoid secretion. 

Previous studies are consistent with our data, in which clinical assays have shown changes in the balance of pro- and anti-inflammatory cytokines in patients with MDD without pharmacological treatment [21] and lower IFN-*γ* and IL-2 levels [52]. Moreover, suicidal depressed patients have a proinflammatory cytokine profile, whereas no such patients have an anti-inflammatory profile [53]; in our study, an exclusion criterion was the presence of suicidal ideation. Notably, *in vitro* studies have reported that high levels of anti-inflammatory cytokines, such as IL-4, are associated with elevated cortisol and can impede the capture of 5-HT [32], significantly downregulating cytokines, such as IL-2 and IFN-*γ*, in MDD patients [21], acting primarily through immunological antagonism between pro- and anti-inflammatory cytokines. 

Antidepressants increased serotonin levels in MDD patients, inducing clinical remission at W20, as assessed by HDRS and BDI scores. At this time point, IL-2 and IFN-*γ* levels rose significantly compared with W0 although there were no substantial changes in cortisol levels between SSRIs alone and SSRIs plus hDLEs, which increased the levels of these cytokines and decreased cortisol levels; this reduction was maintained until the end of treatment. The rise in proinflammatory cytokines might be attributed to the immunostimulatory action of serotonin. Studies have demonstrated that the effects of serotonin on the immune response are dose dependent—proinflammatory cytokine secretion and cellular proliferation occur at physiological serotonin concentrations (0.15 to 1.5 *μ*g/mL of serotonin), whereas such secretion declines at supraphysiological doses (15 *μ*g/mL) [50, 54]. 

These findings contrast with other reports, in which SSRIs decreased proinflammatory cytokine levels in MDD patients [55], which might be due to the time of administration of SSRIs (our study followed up for 52 weeks versus 6 weeks in other studies), the patient demographics (gender, ethnic group, and family history), or depression subtypes [56]. Throughout the administration of SSRIs and SSRIs plus hDLEs, proinflammatory cytokine levels differed in MDD patients. At W20, the SSRI group and SSRI plus hDLE group experienced the largest increase of IFN-*γ* and IL-1*β*; whereas the latter maintained such levels to W52, the levels in the SSRIs group were comparable with those of healthy volunteers. 

In contrast, IL-1*β* levels in the SSRIs group remained higher until the end of treatment, and IL-1*β* decreased, approximating levels in healthy volunteers. IL-2 levels climbed in the SSRI and SSRI plus hDLE groups until W36, which remained elevated until the end of treatment. At W52, the SSRI group had lower IL-1*β* levels versus at W0. 

As discussed, SSRIs increase circulating levels of plasma serotonin [35], the physiological doses of which can upregulate IL-1*β* and IFN-*γ* secretion [50]. This evidence could explain the rise in proinflammatory cytokines in both patient groups. 

DLEs have immunomodulatory and immunostimulatory effects in various infectious diseases, autoimmune diseases, and cancers [57–59]. The coadministration of SSRIs and hDLEs increased IL-1*β*, IL-2, and IFN-*γ* levels. In addition, *in vitro* studies have shown that hDLEs induce IFN-*γ* secretion in Jurkat cells [23]. The mechanism underlying the effects of hEDL still not elucidate, but certain peptides are recognized by innate immune receptors, such as TLRs, on macrophages, B cells, and dendritic cells [60, 61]. These antigen-presenting cells might present hEDL peptides to T cells and induce the release of proinflammatory cytokines.

In our study, MDD patients had an anti-inflammatory cytokine profile that was associated with increased cortisol levels before treatment. Healthy volunteers had undetectable levels of IL-4. SSRIs and SSRIs plus hDLEs downregulated IL-10 and IL-13, but SSRIs failed to reduce the increased levels of IL-4. Notably, *in vitro* studies have linked high levels of anti-inflammatory cytokines, such as IL-4, to elevated cortisol, which can inhibit the capture of 5-HT [32], causing a significant decrease in cytokines, such as IL-2 and IFN-*γ*, in MDD patients. In the SSRI-plus-hDLE group, IL-4 levels were not detected during clinical followup. 

At W52, patients who were given SSRIs and hDLEs experienced a decline in IL-10 and IL-13 to comparable levels in healthy volunteers. In contrast, SSRI-treated patients showed significant variations in IL-10 and IL-13 levels versus healthy volunteers at W52. 

*Limitations.* The limitations of our study are the small sample size and open-label study design without placebo control group. In addition the total scores of the Hamilton Depression Rating Scale (HDRS) showed no significant changes between treatments but will be necessary to analyze if the assessment of each “symptom cluster of HDRS” (anxiety, affective, or somatic symptoms) is associated to molecular variations detected in this study.

## 5. Conclusions

Our report is the first study to analyze the balance between pro- and anti-inflammatory cytokines over 52 weeks of treatment, using an alternative treatment to the classical pharmacologic regimen of SSRIs. hDLEs potentiate the effects of SSRIs on HPA axis hyperactivity by decreasing cortisol levels early in the course of treatment; at the end of the study, patients who were treated with SSRIs and hDLEs consistently had a mixed pro- and anti-inflammatory cytokine pattern. Further studies with more MDD patients are necessary to determine the significance of these findings and their clinical implications for the development of alternative therapeutic approaches in the treatment of major depression. 

## Figures and Tables

**Table 1:** Demographic characteristics in depressed subjects and healthy volunteers.

	Age (years)	Gender (male/female)	BMI (kg/m^2^)	Education (years)	Family history (positive/negative)	First episode	Recurrent episode
Healthy volunteers (*n *= 30)	32 ± 6	10/20	24.3 ± 0.4	15 ± 3	NA	NA	NA
Patients/SSRIs (*n *= 31)	35 ± 9	10/21	24.6 ± 0.7	13 ± 2	10/21	15	16
Patients/SSRIs plus DLE (*n *= 34)	33 ± 9	6/28	24.0 ± 4.0	12 ± 3	16/18	22	12

Values are given as mean ± standard deviation. Education refers to the number of years of schooling. Family history is expressed as the number of patients with depressive antecedents (positive) versus the number of patients without depressive antecedents (negative). NA: nonapplicable. BMI: body mass index.

**Table 2:** Hamilton depression rate score in depressive patients.

	W0	W5	W20	W36	W52
Patients/SSRIs (*n* = 31)	20 ± 2 (*n* = 31)	10 ± 2 (*n* = 31)	3.3 ± 2 (*n* = 25)	4 ± 2 (*n* = 21)	2.6 ± 1.9 (*n* = 11)
Patients/SSRIs plus hDLE (*n* = 34)	24 ± 4 (*n* = 31)	13 ± 4 (*n* = 34)	2.8 ± 3 (*n* = 24)	2 ± 1 (*n* = 20)	2.4 ± 1 (*n* = 10)

Values are given as mean ± standard deviation.
